# Supplementary material for: Maternal Immune Activation Induced by Prenatal Lipopolysaccharide Exposure Leads to Long-Lasting Autistic-like Social, Cognitive and Immune Alterations in Male Wistar Rats
Source: Int J Mol Sci. 2023 Feb 15;24(4):3920. doi: 10.3390/ijms24043920 (PMC9968168; doi:10.3390/ijms24043920)
Supplement: Supplementary file 1 [file ijms-24-03920-s001.zip › ijms-2185870-supplementary.pdf]

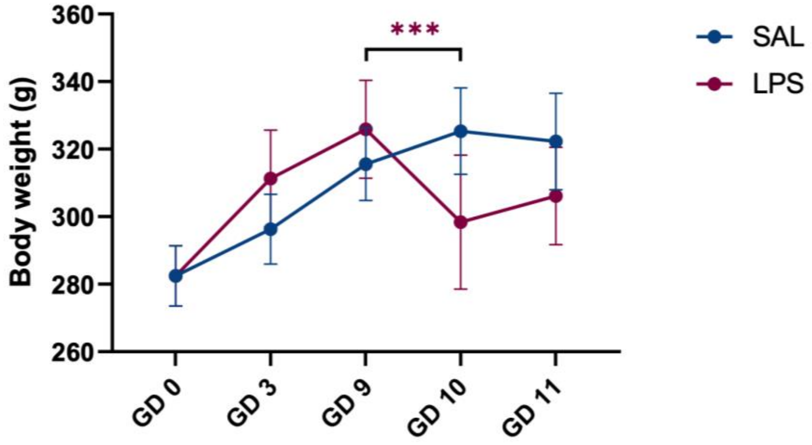

Figure S1. Body weight of the dams before and after SAL or LPS administration. Pregnant rats treated with LPS showed, at GD 10, a significant loss of weight compared to GD9 (SAL = 9, LPS = 8). Data represent mean ± S.E.M. *** $p < 0.001$ vs. LPS GD 9 (Student's *t*-test).

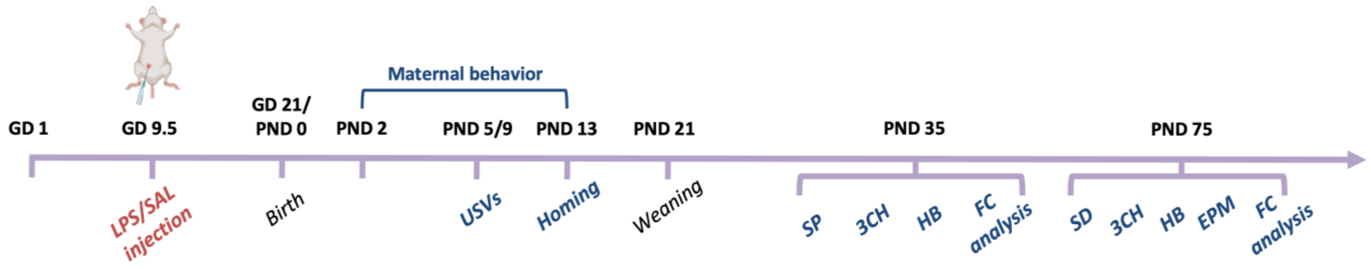

Figure S2. Timeline of experiments.
